# Viewpoint of a WHO Advisory Group Tasked to Consider Establishing a Closely-Monitored Challenge Model of COVID-19 in Healthy Volunteers

**DOI:** 10.1093/cid/ciaa1290

**Published:** 2020-08-28

**Authors:** Myron M Levine, Salim Abdullah, Yaseen M Arabi, Delese Mimi Darko, Anna P Durbin, Vicente Estrada, Euzebiusz Jamrozik, Peter G Kremsner, Rosanna Lagos, Punnee Pitisuttithum, Stanley A Plotkin, Robert Sauerwein, Sheng-Li Shi, Halvor Sommerfelt, Kanta Subbarao, John J Treanor, Sudhanshu Vrati, Deborah King, Shobana Balasingam, Charlie Weller, Anastazia Older Aguilar, M Cristina Cassetti, Philip R Krause, Ana Maria Henao Restrepo

**Affiliations:** 1 Center for Vaccine Development and Global Health, University of Maryland School of Medicine, Baltimore, MD, USA; 2 Ifakara Health Institute (IHI), Ifakara, Tanzania; 3 College of Medicine, King Saud Bin Abdulaziz University for Health Sciences, King Abdullah International Medical Research Center, Riyadh, Kingdom of Saudi Arabia; 4 CEO, FDA, Cantonments, Accra, Ghana; 5 Department of International Health, Johns Hopkins University Bloomberg School of Public Health, Baltimore, MD, USA; 6 Medical School, Complutense University, Hospital Clínico San Carlos, Madrid, Spain; 7 Monash Bioethics Centre, Monash University, Victoria, Australia; 8 Institut für Tropenmedizin, Universitätsklinikum Tübingen, Germany; 9 Centre de Recherches Médicales de Lambaréné, Gabon; 10 Centro para Vacunas en Desarrollo, Santiago, Chile; 11 Vaccine Trial Centre, Faculty of Tropical Medicine, Mahidol University, Bangkok, Thailand; 12 Department of Pediatrics, University of Pennsylvania, Doylestown, PA, USA; 13 Medical Parasitology Department, Radboud University, Nijmegen, The Netherlands; 14 Director Center for Emerging Infectious Diseases, Wuhan Institute of Virology, Wuhan, China; 15 Centre for Intervention Science in Maternal and Child Health, Department of Global Public Health and Primary Care, University of Bergen, Bergen, and Norwegian Institute of Public Health, Oslo, Norway; 16 WHO Collaborating Centre for Reference and Research on Influenza and Department of Microbiology and Immunology, University of Melbourne at the Peter Doherty Institute, Melbourne, Australia; 17 Infectious Diseases Division, University of Rochester Medical Center, Rochester NY, USA; 18 Regional Centre for Biotechnology, Faridabad, Haryana (NCR Delhi), India; 19 Vaccines Priority Area, Wellcome Trust, , London, UK; 20 Vaccines, Wellcome Trust, , London, UK; 21 Vaccines Programme, Wellcome Trust, , London, NW1 2BE, UK; 22 Global Health Discovery & Translational Sciences, Bill & Melinda Gates Foundation, Seattle, WA, USA; 23 Division of Microbiology and Infectious Diseases, National Institute of Allergy and Infectious Diseases, NIH, Bethesda, MD; 24 Office of Vaccines Research and Review, CEBR, FDA, Silver Spring, MD, USA, and Chair, WHO R&D Blueprint COVID-19 Vaccines Working Group; 25 Office of the Executive Director (WHE), WHO Health Emergencies Programme, World Health Organization, Geneva, Switzerland

**Keywords:** COVID-19, SARS-CoV-2, challenge model, experimental challenge, adult volunteers

## Abstract

WHO convened an Advisory Group (AG) to consider the feasibility, potential value and limitations of establishing a closely-monitored challenge model of experimental SARS-CoV-2 infection and COVID-19 in healthy adult volunteers. The AG included experts in design, establishment and performance of challenges. This report summarizes issues that render a COVID-19 model daunting to establish (SARS-CoV-2’s potential to cause severe/fatal illness, its high transmissibility, and lack of a “rescue treatment” to prevent progression from mild/moderate to severe clinical illness) and it proffers prudent strategies for stepwise model development, challenge virus selection, guidelines for manufacturing challenge doses, and ways to contain SARS-CoV-2 and prevent transmission to household/community contacts. A COVID-19 model could demonstrate protection against virus shedding and/or illness induced by prior SARS-CoV-2 challenge or vaccination. A limitation of the model is that vaccine efficacy in experimentally challenged healthy young adults cannot *per se* be extrapolated to predict efficacy in elderly/high-risk adults.

## Introduction

Recognizing the helpful role that experimental challenge studies in healthy adult volunteers have played in the development of certain vaccines [1-15], some have advocated undertaking such studies with virulent SARS-CoV-2 [16-18]. However, several factors warrant that special caution must be taken when working with SARS-CoV-2, including: the severity of COVID-19, as evidenced by its high case-fatality risk in certain sub-populations (elderly, obese, diabetics, hosts with pulmonary and cardiac disease); severe disease requiring ventilator support, thromboembolic events and deaths (albeit relatively uncommon) also occur in young adults (although risk factors for these outcomes remain uncharacterized); SARS-CoV-2's high transmissibility from person-to-person directly by respiratory droplets and at further distances by airborne droplet nuclei [19]; SARS-CoV-2's ability to remain viable on fomites for hours; since the pandemic began, multiple new clinical presentations of COVID-19 have been described. Finally, as of mid-July 2020, a reliable "rescue treatment" has yet to be identified that can predictably arrest the progression from mild/moderate COVID-19 to serious, life-threatening, illness. Understandably, among experienced challenge model investigators the topic of undertaking challenge studies with virulent SARS-CoV-2 has generated discussion about whether the conditions can be assured to perform challenge studies safely, and what the priority goals should be for such studies.

Taking into account the cited reasons for caution, if conditions were deemed suitable to undertake development of a closely-monitored SARS-CoV-2 challenge model in healthy young adult volunteers, important information could accrue such as: determining whether an initial challenge infection confers significant protection against a subsequent challenge with homologous virus (and whether infection-derived protection extends to other virus clades); identifying potential immunologic correlates of protection against illness and virus shedding that might accompany recovery from a prior SARS-CoV-2 experimental challenge; estimating the efficacy of vaccine candidates based on different vaccine platforms (mRNA, DNA, protein, viral-vectored, inactivated whole virus, live attenuated virus) in preventing COVID-19 illness and SARS-CoV-2 shedding.

In April 2020, the WHO convened a multidisciplinary, multi-continent, group to discuss the concept of volunteer challenges with SARS-CoV-2 from different perspectives. This Advisory Group (AG) included experts in: design and performance of many types of volunteer challenge studies; SARS-CoV-2 virology; measurement of human immune responses to SARS-CoV-2 and other pathogens; clinical management of COVID-19 in diverse settings; regulatory considerations associated with testing and emergency pre-licensure use of vaccines and with larger-scale post-licensure deployment; and Good Manufacturing Practices (GMP) manufacture of viruses. The AG was divided into four subgroups to Clinical Trials Issues, Challenge Virus Strain Issues, Measurement of Immune Responses, and Detection of SARS-CoV-2 in Clinical Specimens. The AG agreed to follow the evaluations of potential treatments aiming to interrupt the progression of COVID-19 from mild/moderate to severe illness, even as it diligently undertook to identify the myriad of technical issues that must be addressed to establish a challenge model (Figure 1). Herein the AG describes a technical roadmap of what needs to be done to initiate a closely-monitored challenge model of SARS-CoV-2, if conditions were deemed appropriate. The AG was instructed not to focus on ethical issues being addressed by another AG [20].

**Clinical Issues**

To minimize the risk to volunteers, the AG recommended that only subjects 18 – 25 years of age without underlying health issues associated with more severe COVID disease (diabetes, pre-diabetes, obesity, cardiovascular disease, etc.) be enrolled. Volunteers should be followed up for at least a year following challenge to ensure any long-term consequences to challenge are not missed. To address SARS-CoV-2's high transmissibility and how challenges might proceed when there is little or no ongoing transmission in a community, the AG recommended that early (**STAGE 1**) doseescalation studies should be performed in High-Level Isolation Units (HLIU) that certify rigorous physical and biological containment [21-23], while assuring facile access/transport to intensive care for volunteers, if necessary. A protocol synopsis incorporating these concepts and a consent form are provided in Supplementary Material.

To protect household and community contacts of challenged volunteers, the AG recommends that these studies, in coordination with local public health and civil authorities, be performed under legal quarantine (health authority-issued state of compulsory isolation) [24, 25]. This is analogous to the compulsory isolation in healthcare facilities of patients with Ebola, MERS, or extensively drug-resistant tuberculosis, until they are no longer infectious, as has occurred under revised isolation/quarantine laws enacted in many countries (and state and municipal jurisdictions therein) in recent years. The precedent for quarantine/compulsory isolation during volunteer challenges was set during early cholera challenges performed with community volunteers at the University of Maryland 's Center for Vaccine Development in Baltimore, MD, in the mid-1970s [1, 26]. Following this approach, a volunteer who wishes to leave the study after it begins, as is their right, could do so (no more study procedures, etc.) but they wouldn't be allowed to leave the Isolation Unit until they were no longer infectious. For quarantine/compulsory isolation studies, volunteers must be stringently screened to enroll only those deemed diligent and committed and who clearly understand this concept. Compulsory isolation/quarantine is distinct from housing volunteers in a high containment facility but allowing them to leave the study prematurely if they agree to continuing follow-up thereafter [27].

To minimize the chance of virus reaching the lungs, the AG recommends that the virus inoculum be instilled into the nostrils of the volunteer (0.5 ml per nostril) using a pipette or a well-characterized nasal spray device that can assure that particle size always exceeds 5 microns in diameter. The AG concluded that initially the steps of dose preparation and intranasal administration of challenge virus to volunteers should be performed in a HLIU with rigorous safeguards against droplet and droplet nuclei airborne transmission to minimize the risk of virus spread to research staff and the community. The AG proposes that ~1x$10^2$, ~1x$10^3$, and ~1x$10^4$ median tissue culture infectious doses ($TCID_{50}$) should be the initial dose levels to be investigated in different groups of volunteers in dose-escalation fashion to achieve a 70% clinical attack risk for mild upper respiratory illness, accompanied by shedding of SARS-CoV-2. There is no way to predict whether multiple passages in tissue culture during manufacture will have attenuated the challenge viruses or whether, in contrast, illness in some volunteers may become severe, an outcome to be avoided. A Data Safety Monitoring Board should review safety and shedding data from all volunteers at each dose level and advise of their decision to recommend, or not, escalation to the next higher dose. Volunteers will remain on the HLIU until they have exceeded the usual upper range of incubation (~14 days) and have ceased shedding virus (confirmed by RT-PCR) for three consecutive days. If stepwise dose-escalation studies investigating different SARS-CoV-2 clades yield a safe model, **STAGE 2** studies involving larger numbers of volunteers could proceed, such as challenge/re-challenge studies to assess the protection against illness and virus shedding conferred by primary SARS-CoV-2 infection and randomized, placebo-controlled assessments of vaccine-induced protection against illness and virus shedding. Challenged volunteers should be followed for at least 12 months to rule out late adverse consequences.

**Selecting challenge virus strains and Biosafety Level-3 (BSL-3) GMP manufacturers**

In case virus growth or yields differ, the AG concluded that two separate isolates should be selected from clade B1 (circulating in Europe and the Americas) and two from clade A (original outbreak strain in China) to be sent to manufacturer(s) to prepare Good Manufacturing Practices (GMP) batches. B1 lineage has a mutation in the spike protein (D614G) that may be important, since these variants exhibit increased attachment to the ACE-2 receptor and may manifest enhance transmissibility. Viruses can be selected that harbor the D614G but few other mutations. A list of isolates was assembled to provide potential challenge viruses. Although documenting the clinical history of patients whose virus isolates are selected is not a regulatory requirement, some AG members opined that, ideally, challenge isolates should be obtained from a subject with non-fatal COVID-19 who did not have known risk factors. Using a virus engineered by reverse genetics was also discussed, since a genetic "bar code" could be inserted to tag this virus. While not an immediate option, this should be considered a back-up where use of a genetically modified organism ("GMO") wouldn't evoke regulatory constraints [28].

Each candidate isolate should undergo three rounds of plaque purification in a validated cell line in a BSL-3 facility; 5-10 passages of virus may be necessary to obtain adequate yields. Challenge strains should undergo Next Generation Sequencing (NGS) at the start and end of manufacturing to detect mutations. Some researchers have observed a deletion that removes the furin cleavage site from the spike protein following culture in Vero cells.

Two viruses (at least one clade B1) that provide good yields should be selected for fill and finish of the challenge material batches to prepare clinical study-ready vials containing challenge virus in frozen liquid at ~$10^2$, ~$10^3$, and ~$10^4$ $TCID_{50}$ dose levels. The AG and prospective manufacturers concluded that the preferred formulation and safest presentation would be frozen liquid containing virus within screw-top vials. Lyophilized formulations were deemed undesirable, as they would require a reconstitution step with diluent that would increase biocontainment risk. To assure there is not substantial loss of virus viability/infectivity over time, vials containing the final virus "drug product" must undergo periodic testing to monitor virus titer ($TCID_{50}$ or PFU). An experienced courier service confirmed the details needed to transport vials of SARS-CoV-2 to challenge study sites.

**Measurements of immune responses and virus shedding**

The AG discussed the importance of measuring a wide array of innate, adaptive humoral (serum and mucosal), and cell-mediated immune responses to SARS-CoV-2 (Table 1). Measurements in larger **STAGE 2** studies, such as challenge/re-challenge studies and preliminary assessments of vaccines, may allow identification of immunologic correlates of protection. Methods to monitor virus shedding were also proposed.

**Ability to extrapolate vaccine efficacy in young adults to vaccine performance in the elderly**

Experience with influenza vaccines instructs that it is problematic to extrapolate vaccine efficacy results from young adults to estimate vaccine efficacy/effectiveness in elderly persons. Immunosenescence renders influenza vaccines less immunogenic and less protective in the elderly [29]. To overcome this, vaccines for the elderly have been developed that include 4-fold higher doses of hemagglutinin, or potent adjuvants. Since several COVID-19 vaccine candidates in clinical trials incorporate new technologies/platforms for which licensed vaccines do not yet exist, there is no basis to predict their efficacy in elderly versus younger adults, prior to field trial evaluation.

**Can evidence of vaccine efficacy in young adults in a challenge study accelerate achieving emergency use authorization by regulatory agencies for broader public health deployment of the vaccine?**

The AG sought to separate the vaccine development paradigm classically followed in development of vaccines to prevent endemic infections versus vaccines against Public Health Emergency of International Concern (PHEIC) pathogens. Classical paradigm vaccine candidates are evaluated step-wise through Phase 1, Phase 2 and Phase 3 clinical trials to establish their safety, immunogenicity and efficacy with a final formulation that can be consistently manufactured [30]. This undertaking typically requires >10 years to bring a vaccine to licensure. Related issues include assuring an adequate supply of vaccine, financing to procure doses for target populations, and a delivery strategy and infrastructure to vaccinate targeted populations. Development of vaccines against PHEIC pathogens requires a greatly accelerated process that overlaps phases and necessitates enhanced coordination among stakeholders.

Heretofore, the paradigm for highly accelerated testing of candidate PHEIC vaccines in clinical trials to show safety, immunogenicity and efficacy leading to pre-licensure emergency use was set during the West African Ebola epidemic with the VSV-vectored Ebola vaccine expressing Ebolavirus Zaire glycoprotein (rVSVΔG-ZEBOV-GP). A WHO-led investigator consortium accelerated development of what is now the licensed Ebola vaccine Ervebo™ (Merck Vaccines) from the clinical experience of a single vaccinated subject (August 2014) to documentation by June 2015 of that vaccine's efficacy in a cluster-randomized controlled Phase 3 trial, a period of only 10 months [31]. The time from Phase 1 and 2 trial results that established dose-level and immunogenicity of rVSVΔG-ZEBOV-GP [32, 33], until initiation of the field trial to assess efficacy of the vaccine in Guinea was only two months [31]. This included preparing the trial site in Guinea, training clinical, field and laboratory staff in Good Clinical Practices (GCP) [34], arranging trial monitoring [34], and installing on-site data management. The field trial provided evidence of efficacy within four months [31]. Importantly, rVSVΔG-ZEBOV-GP's efficacy trial ensued in a low-income country without a research infrastructure or clinical investigators and staff experienced in GCP [34]. COVID-19 vaccines, in contrast, can be assessed with experienced clinical and laboratory research personnel in high-income and low-to-middle-income, countries (LMICs).

With efficacy demonstrated, rVSVΔG-ZEBOV-GP was used as an investigational product under monitored emergency use to control an Ebola outbreak in Southeast Guinea (2016) [35], and then in Democratic Republic of the Congo (2018) [36]. In 2019 the US Food and Drug Administration and European Medicines Agency licensed Ervebo™.

**Public perception**

The AG discussed the public perception of volunteer challenge studies with SARS-CoV-2. Potential volunteers in the USA and other countries are signing up to a website promoting challenge studies. However, in both high-income and LMICs, segments of the population are already hesitant about some of the safest, most important, vaccines in public health (*e.g.*, measles vaccine) and many have vowed to decline immunization with a COVID-19 vaccine [37-39]. Several AG members cautioned that challenge studies undertaken in the absence of an effective "rescue treatment" could incite the anti-vaccine movement and discourage persons with hesitancy toward vaccines from being vaccinated [40], particularly if there is an impression that challenge studies were intended to be a "shortcut". The public trust needed to achieve high vaccination coverage with COVID-19 vaccines could be undermined if there was a highly-publicized serious adverse event in a challenged volunteer [40].

**AG Recommendations**

1. Clinical trials to establish a model of COVID-19 should be divided into an incremental strategy in which STAGE 1 encompasses early studies that explore the model through first-in-human, stepwise, dose-escalation studies with three different dose levels and close monitoring of the volunteers to reveal the clinical response and the virus shedding pattern. Subsequent STAGE 2 studies involving larger numbers of volunteers would address questions such as the level of protection conferred by infection-derived immunity and the preliminary efficacy of different vaccines.
2. Volunteers should be restricted to healthy individuals 18-25 years of age, as these have a much lower case-fatality risk than older COVID-19 patients.
3. To address the high transmissibility of SARS-CoV-2 and the need to administer the virus to volunteers intranasally in a high level of containment that minimizes consequences of droplet and aerosol generation, and to protect clinical research and ancillary staff, STAGE 1 studies to establish the model should be performed in HLIUs, *i.e.*, high-level clinical containment facilities.

4. To allow challenge studies to proceed during periods when there is little or no COVID-19 in the community, and to protect household contacts and community contacts of challenged volunteers, the HLIU for STAGE 1 studies should be placed under legal quarantine/compulsory isolation during the period of the study. If so, a participating volunteer who decides to "leave the study", which is their right, will nevertheless not be allowed to leave the quarantined Isolation Unit until they are no longer infectious. This will require close coordination with local public health and civil authorities where the HLIU is located. The precedent for establishing quarantine was set during early cholera challenge studies in community volunteers performed in USA in the mid-1970s.
5. The AG recommends selecting two isolates from Clade B1 and two from Clade A to send to a GMP manufacturer to have batches of virus prepared in appropriate formulation and presentation for use in a SARS-CoV-2 challenge model.
6. The four selected viruses should be sent to a GMP manufacturer with BSL-3 capability where the viruses would be plaque-purified thrice in qualified cells and sequenced by Next Generation Sequencing before and after manufacture; two GMP batches (at least one clade B1) should be finished and filled to produce vials of the frozen liquid formulation at the three dose levels. The virus titer stability of these challenge products should be monitored over time.
7. Dose levels proposed for the STAGE 1 first-in-human, stepwise, dose-escalation studies of each virus are ~$10^2$, ~$10^3$ and ~$10^4$ $TCID_{50,}$. If necessary, a 10-fold higher dose level, ~$10^5$ $TCID_{50}$, may be prepared.
8. Various therapeutic regimens for COVID-19 that are being tested in large randomized, controlled clinical trials worldwide should be closely followed to see if an intervention emerges that might serve as a credible "rescue treatment" for SARS-CoV-2 volunteer challenge studies to reliably interrupt the progression from mild to severe COVID-19.

Whereas the votes of the AG members on the above-mentioned eight technical recommendations were either unanimous or near unanimous, the AG was split approximately in half in voting their opinions on the three questions shown below.

1. Should challenge studies begin if properly formulated challenge viruses in the three desired dose levels become available in the next few months but there is not yet a recognized "rescue treatment" to arrest the progression of COVID-19 from mild/moderate to severe illness? (10 voted "to begin" without such treatment, 9 voted "not to begin").
2. Will efficacy results in young adults in a challenge model predict efficacy in elderly and high-risk adults? (8 opined the model would and 11 declared it would not).
3. Would challenges in young adult volunteers accelerate the timeline for progressing a vaccine to achieve emergency use authorization for deployment in segments of the population suffering high mortality (elderly, diabetics, etc.), compared to the performance of large-scale randomized, controlled field trials of efficacy that included high-risk target populations? (9 opined challenges would accelerate; 9 thought field trials would be faster; one abstained).

**ACKNOWLEDGMENTS**

We acknowledge the administrative and technical assistance of Ximena Riveros Balta and Neddy Mafunga of the World Health Organization Secretariat. The views presented in this article are those of the authors and do not necessarily reflect those of the U.S. Food and Drug Administration or the National Institutes of Health.

**FUNDING**

No grants or payments of any kind supported the World Health Organization Advisory Group activities.

**POTENTIAL CONFLICTS OF INTEREST**

S.P. reports serving as a consultant to vaccine manufacturers, outside the submitted work. None of the other authors report any conflicts of interest.

**Figure 1 legend.**

Figure 1, discussed at the initial videoconference meeting of the Advisory Group (AG) on April 30, 2020 provides an overview of some the strategic steps and decision trees that the AG agreed to grapple with in considering the feasibility of establishing a closely-monitored experimental challenge model of SARS-CoV-2 virus infection and COVID-19 in volunteers. The first was to select whether to begin with a putatively attenuated SARS-CoV-2 strain or with virulent SARS-CoV-2. Since the AG was unaware of an attenuated strain having progressed to where it could be administered in clinical trials, discussion thereafter focused on issues associated with challenge of volunteers with virulent SARS-CoV-2. Several AG members were concerned that clinical studies should not begin until there was a proven “rescue treatment” efficacious in reliably arresting the progression of COVID-19 illness from a mild/moderate status to severe COVID-19. While that “gate” remained in the background, the AG agreed to follow the progress of therapeutic regimens that were in controlled clinical trials to identify a “rescue treatment”. During the months that the AG was active (until early June 2020), remdesivir was reported to diminish the days of hospitalization of severe COVID-19 cases and subsequently dexamethasone was shown to diminish mortality of hospitalized patients. However, neither of these constitute a “rescue treatment” defined as a specific treatment capable of reliably interrupting the progression of mild/moderate COVID-19 to severe illness.

The AG discussed two main uses for a SARS-CoV-2 challenge model once the initial dose/escalation was completed and an acceptable, predictable challenge dose was identified that could be used to answer specific questions. One was re-challenge of a group of volunteers who shed SARS-CoV-2 and developed mild illness on an initial challenge ~6 weeks earlier, along with a new group of naïve control volunteers. Such studies could explore whether the immune responses elicited in the re-challenged “veteran” volunteers may be reflective of protection, as evidenced by diminished shedding of SARS-CoV-2 and prevention of clinical COVID-19 upon re-challenge. If substantial protection was observed it would be possible to look for an immune response (e.g., IgG anti-spike receptor binding domain antibodies, or neutralizing antibodies) that correlated with protection. The other main use of the model, once established, would be to assess preliminarily the efficacy of COVID-19 vaccines based on somewhat different concepts. Evidence of protection of subjects given COVID-19 vaccines against challenge with virulent SARS-CoV-2 could set the stage for identifying correlates of protection, as the serum and mucosal antibodies and cell-mediated immune response measurements would be available from pre- and post-vaccination and from immediately pre- and post-challenge specimens. If significant protection was observed against both clinical endpoints and against virus shedding, this information would contribute to the development of efficacious COVID-19 vaccines by helping to elucidate how they function, based on data generated under closely monitored experimental conditions.

**Table 1. Immunologic assays to be considered during COVID-19 challenge studies**

| Immune effector | Clinical specimen | Antigen (source) | Measure | Timepoints | Assay(s) | Comments |
|---|---|---|---|---|---|---|
| Antibodies | Serum/plasma | Spike or S1 protein or receptor binding domain (RBD) | Binding | Baseline, days 7, 14, 21 and 28 | IgG, IgM and IgA ELISA; IgG subclasses | Binding antibody; subclasses |
| | Serum/plasma | Live (infectious) virus | Neutralizing Ab | Baseline, days 7, 14, 21 and 28 | Neutralizing Ab | Requires BSL3 containment; gold standard |
| | Serum/plasma | Pseudovirus expressing SARS-CoV-2 spike protein | Neutralization of virus entry | Baseline, days 7, 14, 21 and 28 | Neutralizing Ab | Must be accompanied by neutralization assays using infectious virus |
| | Serum/plasma | Surrogate sVNT | Competitive ELISA for RBD binding to ACE2 | Baseline, days 7, 14, 21 and 28 | Surrogate for virus neutralization | |
| | Serum | Spike or S1 protein | Antibody dependent cell cytotoxicity (ADCC) | Baseline, days 7, 14, 21 and 28 | ADCC | |
| | Nasal wash | Spike or S1 protein | Mucosal antibodies: IgG and IgA including IgA subclasses | Baseline, days 7, 14, 21 and 28 | ELISA | |

| | | | | | | |
|---|---|---|---|---|---|---|
| Cellular | Whole blood or peripheral blood mononuclear cells (PBMC) | | Counts of B and T (CD4+ and CD8+) cells | Baseline, days 7, 14 and 28 | Flow cytometry | |
| | Whole blood or peripheral blood mononuclear cells (PBMC) | SARS-CoV-2 peptides or inactivated virus | cTfh; Activated CD8+ T cells; B cells: Antibody secreting cells (ASC); memory B cells | Baseline, days 7, 14 and 28 | Flow cytometry | |
| | Peripheral blood mononuclear cells (PBMC) | S1 protein or SARS-CoV-2 peptides or inactivated virus | B cells: IgG, IGA and IgM antibody secreting cells (ASC) | Baseline, days 7, 14 and 28 | ELISPOT | |
| | Peripheral blood mononuclear cells (PBMC) or serum or plasma | S1 protein or SARS-CoV-2 peptides or inactivated virus | Cytokines | Baseline and every other day | Cytokine assays | Th 1/2 orientation; Th17 cells and IL-17 production |
| Innate immune system | Serum | N/A | CRP protein level | Baseline and Day 1 and 3 minimum | CRP protein | |
| Transcriptomics | Whole blood PAXgene tubes | N/A | Transcriptomics | Baseline and every other day | Transcriptomics | |

Figure 1

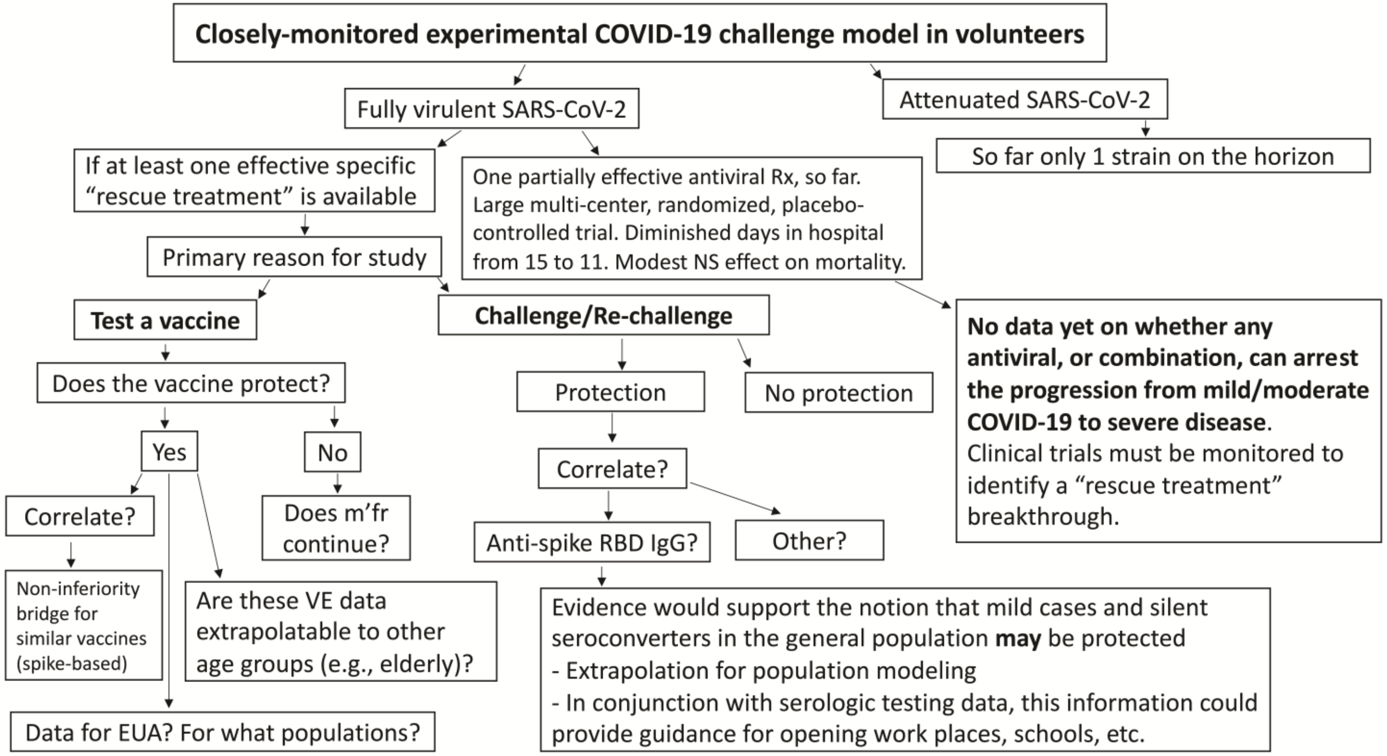

Closely-monitored experimental COVID-19 challenge model in volunteers
Fully virulent SARS-CoV-2
Attenuated SARS-CoV-2
So far only 1 strain on the horizon
If at least one effective specific "rescue treatment" is available
One partially effective antiviral Rx, so far. Large multi-center, randomized, placebo-controlled trial. Diminished days in hospital from 15 to 11. Modest NS effect on mortality.
Primary reason for study
Test a vaccine
Challenge/Re-challenge
No data yet on whether any antiviral, or combination, can arrest the progression from mild/moderate COVID-19 to severe disease. Clinical trials must be monitored to identify a "rescue treatment" breakthrough.
Does the vaccine protect?
Protection
No protection
Yes
No
Correlate?
Does m'fr continue?
Correlate?
Anti-spike RBD IgG?
Other?
Non-inferiority bridge for similar vaccines (spike-based)
Are these VE data extrapolatable to other age groups (e.g., elderly)?
Evidence would support the notion that mild cases and silent seroconverters in the general population may be protected
- Extrapolation for population modeling
- In conjunction with serologic testing data, this information could provide guidance for opening work places, schools, etc.
Data for EUA? For what populations?
